# Pexidartinib treatment in Alexander disease model mice reduces macrophage numbers and increases glial fibrillary acidic protein levels, yet has minimal impact on other disease phenotypes

**DOI:** 10.1186/s12974-021-02118-x

**Published:** 2021-03-08

**Authors:** Michelle M. Boyd, Suzanne J. Litscher, Laura L. Seitz, Albee Messing, Tracy L. Hagemann, Lara S. Collier

**Affiliations:** 1 Pharmaceutical Sciences Division, School of Pharmacy, University of Wisconsin-Madison, Madison, USA; 2 Waisman Center, University of Wisconsin-Madison, Madison, USA; 3 Department of Comparative Biosciences, School of Veterinary Medicine, University of Wisconsin-Madison, Madison, USA

**Keywords:** Pexidartinib, CSF1R, Alexander disease, GFAP, Macrophage

## Abstract

**Background:**

Alexander disease (AxD) is a rare neurodegenerative disorder that is caused by dominant mutations in the gene encoding glial fibrillary acidic protein (GFAP), an intermediate filament that is primarily expressed by astrocytes. In AxD, mutant GFAP in combination with increased GFAP expression result in astrocyte dysfunction and the accumulation of Rosenthal fibers. A neuroinflammatory environment consisting primarily of macrophage lineage cells has been observed in AxD patients and mouse models.

**Methods:**

To examine if macrophage lineage cells could serve as a therapeutic target in AxD, GFAP knock-in mutant AxD model mice were treated with a colony-stimulating factor 1 receptor (CSF1R) inhibitor, pexidartinib. The effects of pexidartinib treatment on disease phenotypes were assessed.

**Results:**

In AxD model mice, pexidartinib administration depleted macrophages in the CNS and caused elevation of GFAP transcript and protein levels with minimal impacts on other phenotypes including body weight, stress response activation, chemokine/cytokine expression, and T cell infiltration.

**Conclusions:**

Together, these results highlight the complicated role that macrophages can play in neurological diseases and do not support the use of pexidartinib as a therapy for AxD.

**Supplementary Information:**

The online version contains supplementary material available at 10.1186/s12974-021-02118-x.

## Introduction

Alexander disease (AxD) is a rare neurological disorder that generally results in neurodegeneration and death. AxD is caused by dominant mutations in the glial fibrillary acidic protein (GFAP) gene that result in astrocyte dysfunction and ultimately other disease phenotypes. The hallmark pathology observed in AxD is the presence of Rosenthal fibers that are cytoplasmic protein aggregates in astrocytes that contain GFAP and other proteins [13]. Genetic mouse models have been generated to study disease processes and potential treatment strategies. These models include transgenic over-expression of human *GFAP* (*GFAP*^Tg^), engineering of human disease-causing point mutations into the endogenous *Gfap* locus (e.g., *Gfap*^R236H/+^), and a severely affected model that combines the two (*GFAP*^Tg^;*Gfap*^R236H/+^) [8, 19]. A neuroinflammatory response primarily comprised of cells expressing the pan-macrophage marker IBA1 has been observed in the brains of human patients as well as mouse models for AxD, though the role that this inflammatory response plays in AxD is not known [9, 10, 21].

Interest is mounting in studying the role of macrophage lineage cells in the pathogenesis of other neurodegenerative disorders (reviewed in [22, 23]). However, the role of macrophages in a primary astrocyte disorder such as AxD has not been thoroughly studied. Activated astrocytes, including dysfunctional astrocytes found in AxD, can produce factors that signal to macrophages [17, 21]. In homeostatic conditions, microglia are resident CNS parenchymal macrophages that serve immune and other functions. Under pathologic conditions in the CNS, microglia and infiltrating macrophages can dynamically respond to threats by changing their phenotype, producing cytokines and chemokines, and proliferating (reviewed in [2]).

Colony-stimulating factor 1 receptor (CSF1R) signaling regulates macrophage proliferation, differentiation, and survival ([1, 3, 5], reviewed in [14]). CSF1R has two ligands, colony-stimulating factor 1 (CSF1) and IL-34. The expression of these ligands, especially CSF1, is often upregulated in CNS disease states and injury [7, 27]. Small molecule inhibitors of the tyrosine kinase activity of CSF1R have been used to deplete macrophages and curb neuroinflammation in mouse models of other neurodegenerative diseases [20]. In these studies, CSF1R inhibitors have had both beneficial and harmful effects [20, 28], indicating that the effects of neuroinflammation are context dependent.

In this study, the role of macrophage lineage cells as well as their potential as therapeutic targets for AxD were examined by treating *Gfap*^R236H/+^ AxD model mice with the CSF1R inhibitor pexidartinib (formerly PLX3397, Plexxikon Inc.), which was recently approved by the FDA for treatment of tenosynovial giant cell tumors. Further, the effects of pexidartinib treatment on reported phenotypes in AxD model mice were assessed. We found that pexidartinib administration to AxD model mice caused decreased macrophage numbers and increased GFAP protein levels with minimal impacts on other disease phenotypes.

## Materials and methods

### Ethics statement

Animal experiments were conducted in accordance with the United States National Research Council’s Guide for the Care and Use of Laboratory Animals and under the approval of the Institutional Animal Care and Use Committee at the University of Wisconsin-Madison.

### Mouse experiments

*Gfap*^R236H/+^ (AxD) mice [8] and littermate *Gfap*^+/+^ (wild-type (WT)) mice were congenic on the C57BL/6J background (>20 generations of backcrossing). As *Gfap*^R236H/+^ mice age, GFAP levels in the brain increase [8]. Therefore, to determine if pexidartinib (PEX) treatment prevents this progressive GFAP accumulation and ameliorates other disease phenotypes, drug treatment began at weaning (postnatal day 21) and mice were treated into adulthood (2.5 months of treatment). Mice were treated with PEX provided by Plexxikon Inc. in AIN-76A rodent diet (275 mg/kg chow) or just AIN-76A diet (control, CON) by Research Diets. Littermates were split across treatments to avoid any litter specific issues. Mice were housed with cage companions in a specific pathogen free environment under a 12-h light/dark cycle with food and water provided ad libitum. Mice were weighed weekly throughout the study. At study endpoint, mice were euthanized with pentobarbital and perfused transcardially with phosphate-buffered saline (PBS). Brains used for histological staining were post-fixed in 4% paraformaldehyde (PFA) overnight followed by cryoprotection in sucrose prior to sectioning with a cryostat, with the exception of brains used for Rosenthal fiber analyses which were post-fixed overnight in methacarn fixative prior to paraffin embedding. Brain regions used for RT-qPCR and ELISA were dissected and snap frozen in liquid nitrogen. All analyses included equal (when *N*s are even numbers) or approximately equal (when *N*s are odd numbers) numbers of male and female mice, except for Rosenthal fiber analyses, where groups included both sexes but not in equal numbers.

### GFAP quantification

Snap frozen olfactory bulbs or hippocampi were homogenized in 0.2 ml or 0.35 ml, respectively, of lysis buffer (2% SDS, 50 mM Tris-HCl pH 7.4, 5 mM EDTA pH 7.4, 1 mM PefablocSC (4-(2-Aminoethyl)benzenesulfonyl fluoride hydrochloride, Millipore Sigma, Cat# 11429868001), and 1X complete proteinase inhibitor in water). Samples were then boiled for 15 min and diluted 1:20 in 1X PBS. The Pierce BCA assay using BSA standards (Thermo Cat#23227) was used to determine the total protein concentration in the lysates. An enzyme-linked immunosorbent assay (ELISA) which included GFAP protein standards (Fitzgerald Industries International Inc. #30R-AG009) was used to quantify the amount of GFAP protein as previously described [10]. All samples were analyzed with technical duplicates.

### Quantitative real-time PCR

Total RNA was extracted from dissected olfactory bulbs and hippocampi in Trizol (Ambion Cat# 15596026) following the manufacturer’s recommended protocol (Invitrogen Cat# 12183018A) with on-column DNAse treatment (Invitrogen Cat# 12185-010). cDNA was made using a high capacity cDNA reverse transcription kit (Thermo Cat# 4368814). RT-qPCR was conducted with SYBR green master mix (Applied Biosystems Cat# A25742) on a Step One Plus Real-Time PCR System (Applied Biosystems). Samples for each animal were analyzed as technical duplicates. Data displayed in figures are presented as values normalized to *Tbp* and relative to WT control as 1 unless otherwise noted in the figure legend. Primers (listed in Table 1) used for RT-qPCR assays were efficiency tested on appropriate tissue with known expression.

**Table 1:** RT-qPCR primer sequences

Gene	Forward primer	Reverse primer
*Gfap*	ACATGCAAGAGACAGAGGAGTGGT	AGTCGTTAGCTTCGTGCTTGGCTT
*Il34*	GACGTGGCTTTGGGAAACGAGAAT	AGGCACAGCAATCCTGTAGTTGATGG
*Il6*^a^	TCCTTAGCCACTCCTTCTGT	AGCCAGAGTCCTTCAGAGA
*Csf1*^a^	GGCATCATCCTAGTCTTGCTG	ACCTGTCTGTCCTCATCCT
*Aif1*	TGATGAGGATCTGCCGTCCAAACT	TCTCCAGCATTCGCTTCAAGGACA
*Cryab*^a^	GTCTGACCTCTTCTCAACAGC	ATCTGTCCTTCTCCAAACGC
*Cxcl1*^a^	CCAAACCGAAGTCATAGCCA	GTGCCATCAGAGCAGTCT
*Cxcl10*^a^	ATTTTCTGCCTCATCCTGCT	TGATTTCAAGCTTCCCTATGGC
*Il1b*^a^	CTCTTGTTGATGTGCTGCTG	GACCTGTTCTTTGAAGTTGACG
*Tbp*^a^	TTCACCAATGACTCCTATGACC	CAAGTTTACAGCCAAGATTCACG
*Lcn2*^a^	CTACAATGTCACCTCCATCCTG	CCTGTGCATATTTCCCAGAGT
*Nfe2l2*^a^	TCAAACACTTCTCGACTTACTCC	TGATGGACTTGGAGTTGCC
*Nqo1*^a^	GCCAATGCTGTAAACCAGTTG	GCTCCATGTACTCTCTTCAGG
*Mog*	GCTTCTTCAGAGACCACTCTT	GATAGGCACAAGTGCGATGA
*Plp1*	CCTGTTTATTGCTGCGTTTGT	TAAGGACGGCGAAGTTGTAAC

^a^Denotates primers that were ordered pre-designed from IDT prime-time

### Rosenthal fiber accumulation quantification

Ten-micrometer sections were stained with hematoxylin and eosin and six sagittal ×40 images were taken focusing on the olfactory bulb glomerular layer and along the hippocampal fissure in similar locations. Rosenthal fiber accumulation was assessed in each image by scoring severity on a scale of 0 (none), 1 (mild), 2 (moderate), or 3 (strong) [6], and the median severity score for each animal was calculated for each brain region. The researcher was blinded to the experimental groups for the animals during assessment.

### Immunofluorescence staining

For olfactory bulb, 15 μm sagittal sections were baked at 37 °C onto slides for 30 min. For hippocampus, 40 μm free floating coronal brain slices were used. Following a PBS wash, sections were permeabilized in 0.5% Triton X-100 in PBS for 10 min. Following a PBS wash, sections were blocked in 1% BSA or 5% normal goat serum for 1 h followed by primary antibody incubation for 3 to 4 h at room temperature (olfactory bulb, IBA1 staining) or overnight at 4 °C (all other staining). Antibodies used were commercially validated for mouse immunohistochemistry reactivity. Sections were washed in PBS and then incubated in appropriate secondary antibodies with DAPI (Millipore Sigma Cat# D8417, 1:1,000) for 1 h at room temperature. Slides were mounted in Vectashield (Vector Labs Cat# H-1000). For the olfactory bulb, due to macrophage density IBA1^+^ cells were counted from six images at high magnification (×40 with Nyquist zoom on a Nikon A1R confocal microscope with a 10 μm Z-stack (1 μm step) acquired with NIS-Elements AR Software). All other imaging was done on an Olympus Fluoview FV1000 confocal microscope at ×40 with a 10-μm Z-stack (1 μm step) acquired with FV10-ASW 4.2 software. For IBA1 and CD3 analysis, 5-6 images were used. Image acquisition settings were kept consistent within an experiment across all groups. Images were collapsed into two-dimensional maximum intensity projection images for counting cells. The researcher was blinded to experimental groups for the cell counting analyses. All antibodies used are listed in Table 2.

**Table 2:** Antibodies

Antibody	Dilution	Manufacturer/catalog number	RRID
Rabbit anti-IBA1	1:200	Wako/019-19741	RRID:AB_839504
Rat anti-CD3	1:200	Abcam/Ab11089	RRID:AB_369097
Donkey anti-rabbit 594	1:300	Abcam/Ab150076	RRID:AB_2782993
Goat anti-rat 555	1:125	Thermo/A21434	RRID:AB_2535855

### Statistical analyses

All statistical analyses were performed using GraphPad-Prism and *p*<0.05 was considered statistically significant. For body weight analyses, animals were excluded from analyses if they were missing a body weight from one or more time points throughout the study. For the RT-qPCR experiments, animals were excluded from analyses if the equipment reported a high standard deviation error for technical replicates for that animal. Unless otherwise noted, values shown in all graphs represent the mean ± standard deviation. Statistical analyses and sample sizes are described in figure legends.

## Results

### Increased *Csf1* expression is a consistent feature in AxD model mice

**Fig. 1:**
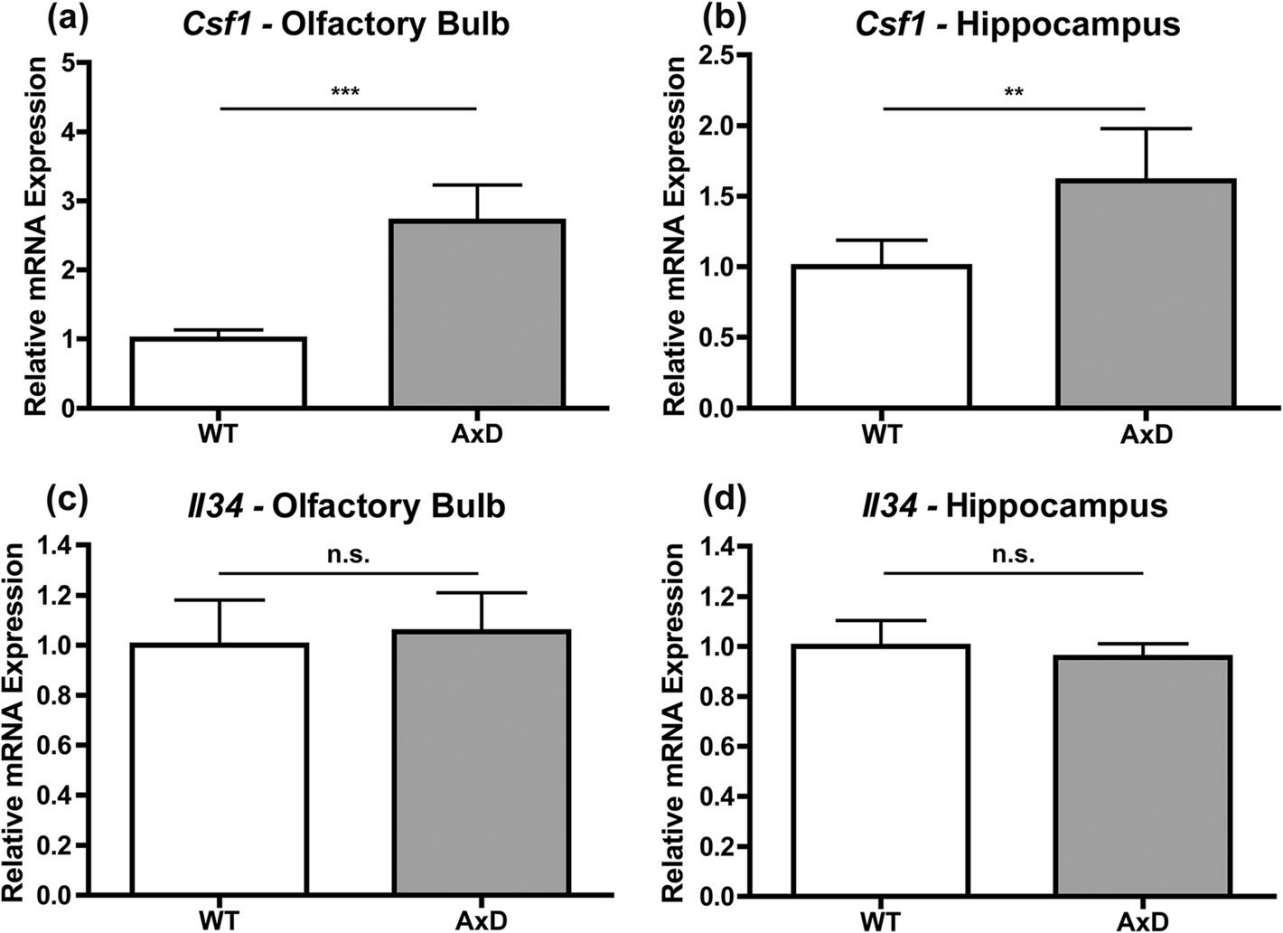
*Csf1* is upregulated in AxD mouse brains. RT-qPCR for *Csf1* for the olfactory bulb (**a**) and hippocampus (**b**) and *Il34* for the olfactory bulb (**c**) and hippocampus (**d**). Statistical analyses: unpaired two-sided *t* tests. n.s., non-significant (*p*>0.05), **p<0.01, ***p<0.001. *N* = 5-6 per group

Increased CSF1 levels were detected in the spinal cord of the *GFAP*^*TG*^ AxD model [21]. However, *Csf1* expression has not been previously investigated in *Gfap*^R236H/+^ mice, and there have been no reports on expression levels of *Il34* in any AxD model. RT-qPCR was therefore used to determine if expression of *Csf1* and *Il34* were upregulated in olfactory bulbs and hippocampi of *Gfap*^R236H/+^ AxD model mice, regions showing GFAP accumulation, astrocyte pathology, and a concomitant macrophage response in *Gfap*^R236H/+^ mice [10, 16]. Compared to WT, AxD mice displayed increased expression of *Csf1* in both the olfactory bulb and hippocampus (Fig. 1a and b). In contrast, expression levels of *Il34* were equivalent in WT and AxD mice (Fig. 1c and d). Given that increased CSF1 expression in the brain is sufficient to increase brain macrophage numbers [3], these data indicate that the CSF1/CSF1R signaling axis is likely contributing to the neuroinflammatory response in AxD models.

### Pexidartinib reduces macrophage numbers in AxD mice

**Fig. 2:**
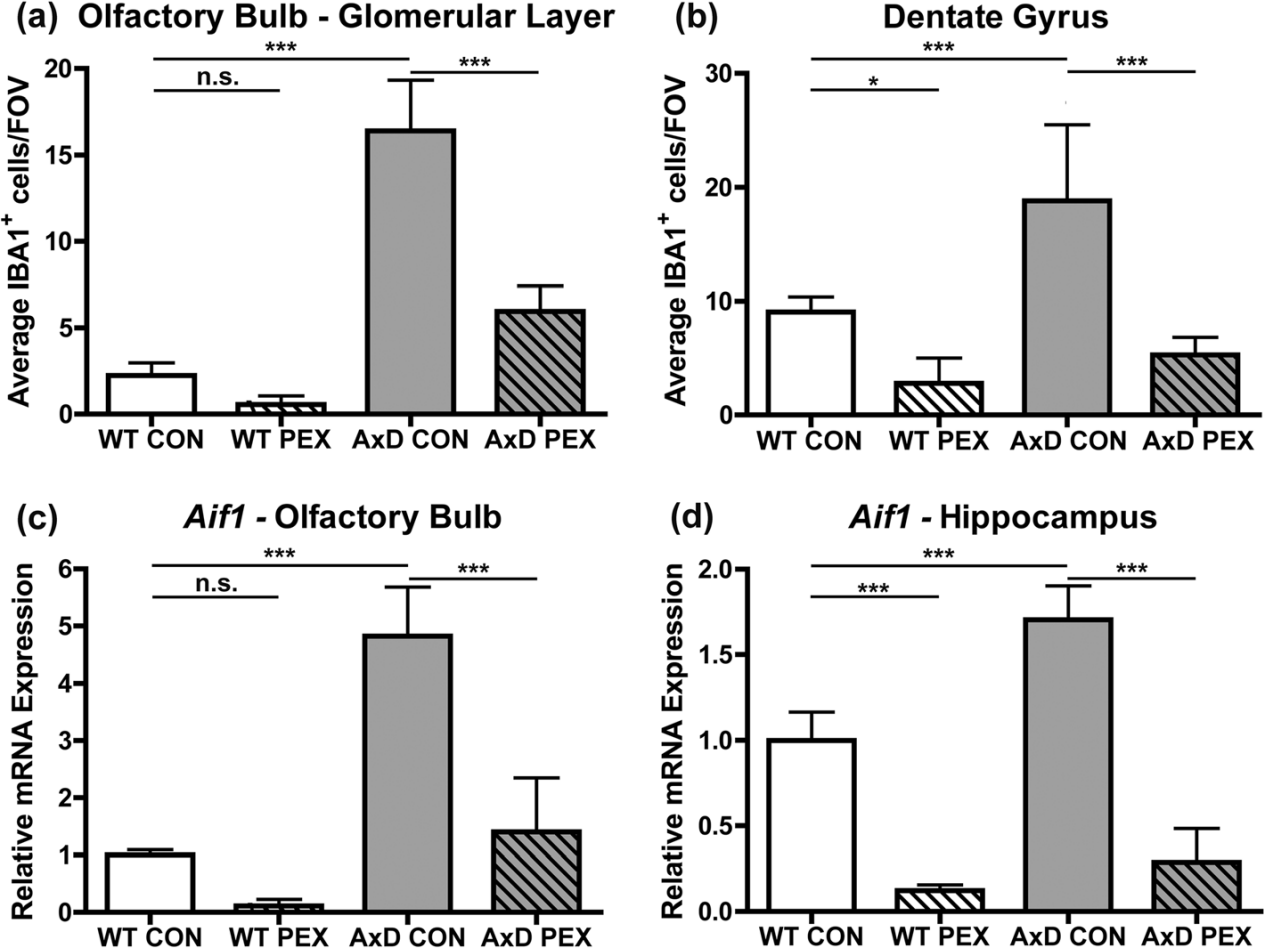
Pexidartinib reduces macrophage numbers in AxD mice. Immunofluorescence was performed for IBA1 and the number of IBA1^+^ cells counted. Data are presented as the average number of cells per field of view (FOV) for the olfactory bulb glomerular layer (**a**) and the dentate gyrus of the hippocampus (**b**). RT-qPCR for *Aif1* (*Iba1*) in the olfactory bulb (**c**) and hippocampus (**d**). Statistical analyses: One-way analysis of variance (ANOVA) with Bonferroni’s post-test for the indicated comparisons. n.s., non-significant (*p*>0.05), *p<0.05, ***p<0.001. *N* =5-8 per group for IBA1 counts. *N* = 5-6 per group for *Aif1* RT-qPCR data

To determine if treatment with the CSF1R inhibitor pexidartinib (PEX) depletes macrophages, we administered the drug via chow fed to *Gfap*^R236H/+^ AxD model mice for a period of 2.5 months, beginning at weaning. Previous reports indicate that PEX treatment can raise GFAP levels in wild-type mice [5]; therefore, wild-type (WT, *Gfap*^+/+^) littermates were also fed PEX or control chow as a comparison. The numbers of macrophages (IBA1^+^ cells) were counted in the olfactory bulb glomerular layer and the dentate gyrus of the hippocampus, areas that have been observed to display Rosenthal fibers in histological analyses [10, 16]. As expected, significant increases in the number of IBA1^+^ cells were observed in both regions in AxD compared to WT animals fed control chow (Fig. 2a and b, representative images: Additional figure 1a and b). PEX treatment of AxD mice resulted in depletion of macrophages in both brain regions (Fig. 2a and b). PEX treatment in WT mice did reduce macrophage numbers in the dentate gyrus, but not in a statistically significant manner in the olfactory bulb glomerular layer (Fig. 2a and b). RT-qPCR for *Aif1* (the gene encoding IBA1) revealed a similar decrease in PEX-treated mice (Fig. 2c and d). Together, these data indicate that PEX treatment reduces brain macrophage numbers in AxD mice and may have region specific effects in WT mice.

### Pexidartinib treatment does not restore body weight in AxD mice

**Fig. 3:**
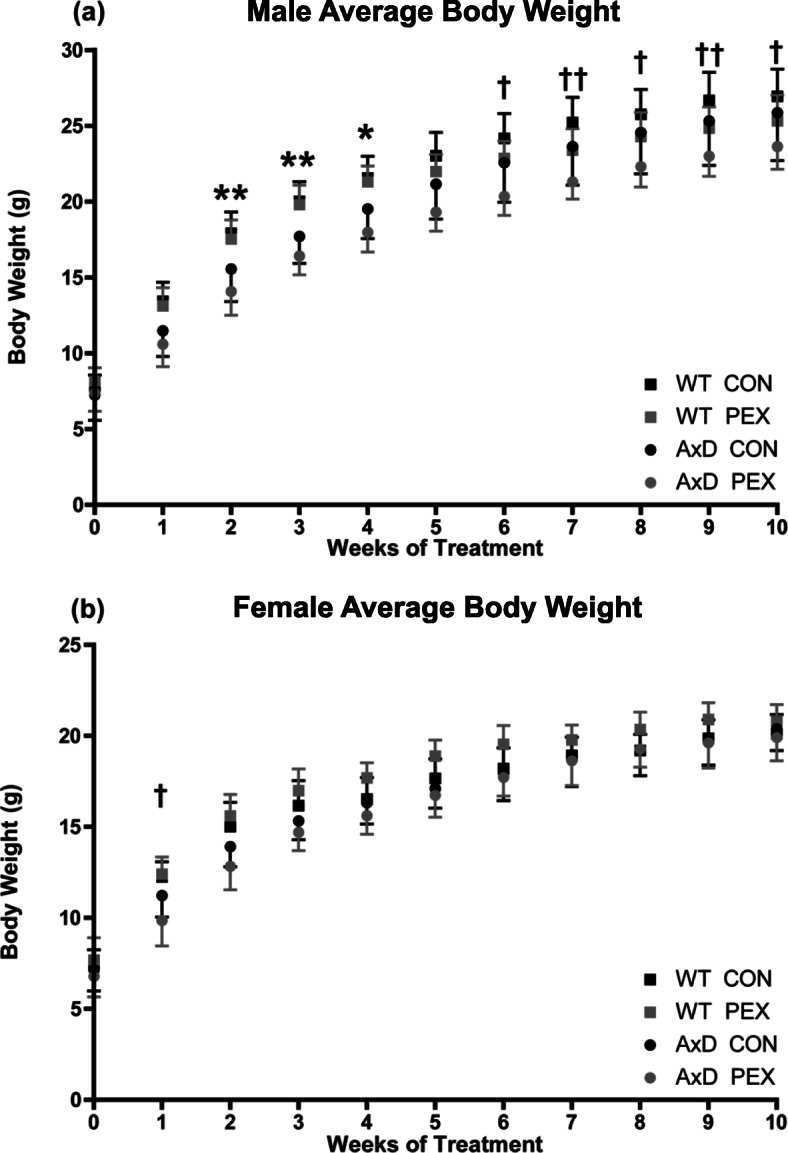
Pexidartinib treatment does not increase body weight in AxD mice. Mice were weighed weekly beginning at treatment initiation (weaning, 3 weeks of age). Due to inherent differences in body weight, males and females were analyzed separately. Weight is expressed in grams (g). (**a**) Average male body weight. (**b**) Average female body weight. Statistical analyses: Two-way repeated measures ANOVA with Bonferroni’s post-test, *p<0.05, **p<0.01 comparisons between WT CON and AxD CON, †*p*<0.05, ††*p*<0.01 comparisons between AxD CON and AxD PEX. *N* = 10-14 per group

To test whether PEX treatment improved the body weight deficit in *Gfap*^R236H/+^ mice [8, 16], animals were weighed weekly throughout the study. Given the conditions of the current study, the weight of AxD male mice fed control chow was only significantly less than WT control treated male mice at weeks 2-4 of treatment, and no difference was observed in female mice at any timepoint (Fig. 3a and b). However, in AxD animals, lower weights were observed with PEX treatment at a limited number of time points (males at weeks 6-10 and females at week 1, Fig. 3a and b). In contrast, PEX did not significantly alter the weight of WT animals (Fig. 3a and b). Collectively, these results indicate that PEX treatment does not improve the body weight phenotype typically associated with AxD mice and at some timepoints actually makes this phenotype worse.

### Pexidartinib treatment of AxD mice results in elevation of GFAP levels

**Fig. 4:**
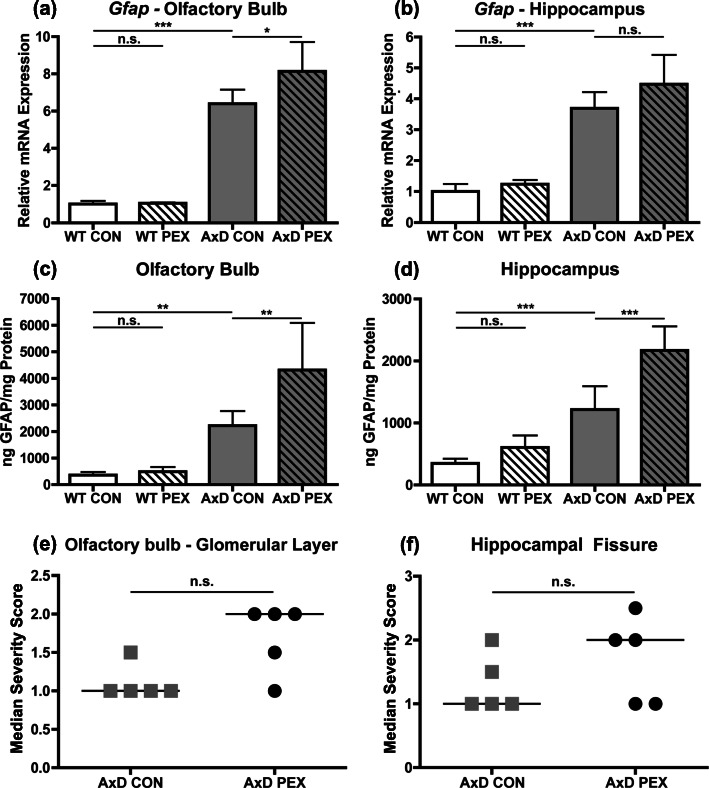
Pexidartinib treatment of AxD mice results in elevation of GFAP levels. RT-qPCR for *Gfap* was performed on mRNA isolated from olfactory bulb (**a**) and hippocampus (**b**). GFAP protein levels in the olfactory bulb (**c**) and hippocampus (d) were measured by ELISA. Rosenthal fiber accumulation was analyzed in the olfactory bulb glomerular layer (**e**) and along the hippocampal fissure (**f**), median shown in graph and horizontal line indicates group median. Statistical analysis: One-way ANOVA with Bonferroni’s post-test for the indicated comparisons for RT-qPCR and ELISAs, Mann-Whitney test for Rosenthal fiber accumulation analyses, n.s., non-significant (*p*>0.05), *p<0.05, **p<0.01,***p<0.001. *N* = 5-6 per group for *Gfap* RT-qPCR and GFAP ELISA. *N* = 4-5 per group for Rosenthal fiber accumulation scoring

*Gfap*^R236H/+^ mice exhibit increased GFAP transcript and protein levels in the olfactory bulb and hippocampus, and Rosenthal fibers accumulate in these regions [8]. In mice given control chow, quantification of *Gfap* and GFAP levels revealed the expected elevation in AxD animals compared to WT in both regions (Fig. 4a-d). With PEX treatment, no change in transcript or protein levels was observed in the WT animals in either brain region (Fig. 4a-d). However, PEX treatment of AxD mice resulted in elevation of *Gfap* transcript in the olfactory bulb and increased GFAP protein in both regions (Fig. 4a-d). Rosenthal fiber accumulation was also assessed in hematoxylin and eosin stained tissue. In both WT groups, no Rosenthal fibers were observed; thus, these groups were omitted from further analyses (data not shown, representative images in Additional figure 2a and b). In AxD mice, PEX treatment did not significantly impact Rosenthal fiber accumulation in either region (Fig. 4e and f, representative images: Additional figure 2a and b). Together, these results indicate that PEX treatment of AxD mice results in elevation of GFAP protein levels with no impact on Rosenthal fiber accumulation. In contrast, GFAP transcript and protein levels were not significantly impacted by PEX treatment in the WT groups.

### Pexidartinib has limited impact on expression levels of astrogliosis and stress response markers in AxD mice

**Fig. 5:**
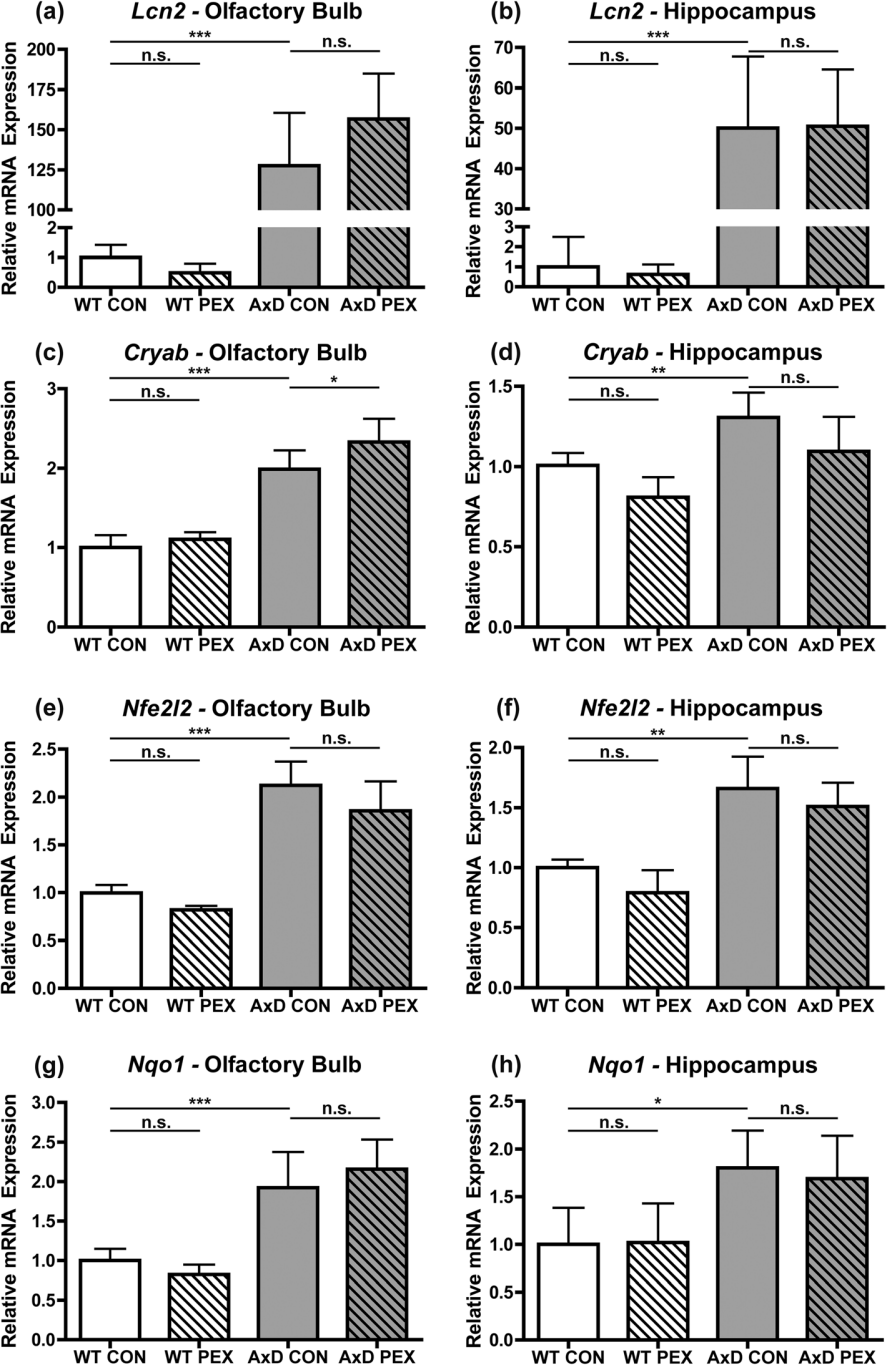
Pexidartinib has limited impact on expression levels of astrogliosis and stress response markers in AxD mice. RT-qPCR for *Lcn2* for the olfactory bulb (**a**) and hippocampus (**b**), *Cryab* for the olfactory bulb (**c**) and hippocampus (**d**), *Nfe2l2 (Nrf2)* for the olfactory bulb (**e**) and hippocampus (**f**), and *Nqo1* for the olfactory bulb (**g**) and hippocampus (**h**). Statistical analyses: One-way ANOVA with Bonferroni’s post-test for the indicated comparisons, n.s., non-significant (*p*>0.05), *p <0.05, **, *p*<0.01, ***p<0.001. *N* = 5-6 per group

Increased expression of several markers of astrogliosis and an accompanying stress response have been previously reported in *Gfap*^R236H/+^ mice [9, 12, 16, 29]. To examine if PEX alters these processes, RT-qPCR was utilized to examine the expression of *Lcn2* (Lipocalin-2), a marker of astrocyte reactivity/astrogliosis; *Cryab*, which encodes a small heatshock protein; *Nfe2l2* (*Nrf2*), a transcription factor responsible for regulating the antioxidant stress response; and *Nqo1*, a downstream target of NRF2. We observed a significant increase in *Lcn2* expression in AxD CON mice in both brain regions; however, there was no change in expression with drug treatment (Fig. 5 a and b). As expected, the reported increase in *Cryab* expression was observed in AxD CON-treated animals compared to WT in both brain regions (Fig. 5c and d). A small but significant increase in *Cryab* expression was observed in AxD PEX-treated mice in the olfactory bulb but not the hippocampus (Fig. 5c and d). The reported increases in *Nfe2l2* and *Nqo1* expression in AxD CON mice were observed in both brain regions (Fig. 5e-h). However, there was no observed change with drug treatment in either brain region of WT or AxD animals for these genes (Fig. 5e-h). Together these results demonstrate that in AxD mice, PEX-mediated increases in GFAP levels are not generally accompanied by increased expression of additional astrogliosis or stress response markers. Additionally, long-term PEX treatment does not cause de novo activation of these responses in WT mice.

### Pexidartinib has limited impact on expression of cytokines and chemokines in AxD mice

**Fig. 6:**
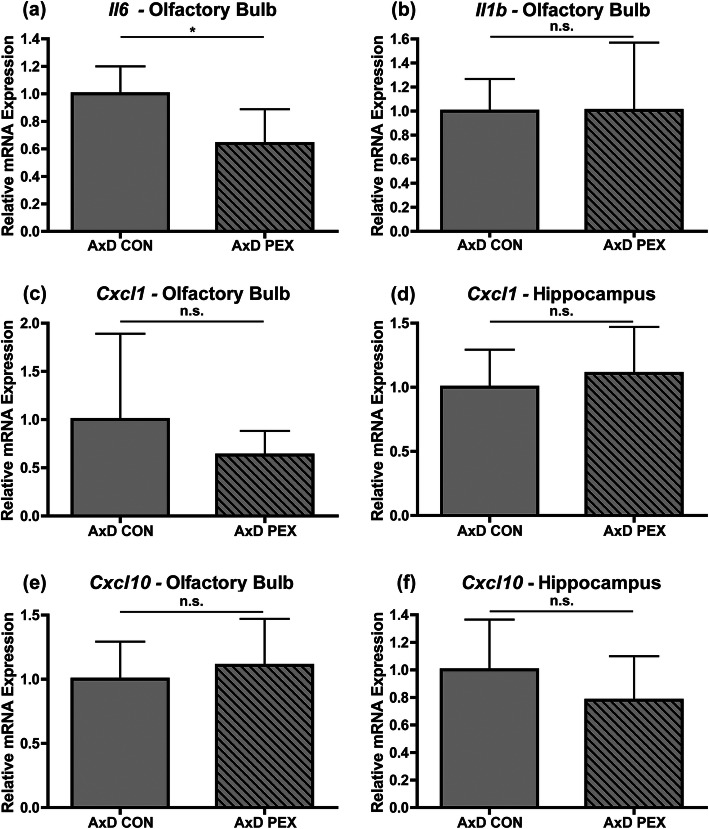
Pexidartinib has limited impact on expression of cytokines and chemokines in AxD mice. RT-qPCR for *Il6* (**a**) and *Il1b* (**b**) was performed on mRNA isolated from olfactory bulb and hippocampus, but only olfactory bulb is shown because expression of these cytokines was not detected in the hippocampus. RT-qPCR for *Cxcl1* for the olfactory bulb (**c**) and hippocampus (**d**), and *Cxcl10* for the olfactory bulb (**e**) and hippocampus (**f**). Data displayed in this figure are presented as values normalized to *Tbp* and relative to AxD control as 1. Statistical analyses: unpaired two-sided *t* test, n.s., non-significant (*p*>0.05), *p <0.05. *N* = 5-6 per group

Increased expression of several cytokines and chemokines have been detected in AxD mouse models, with some being found to be consistently upregulated in all models examined (e.g., *Cxcl1*) while others are only upregulated in a subset of models (e.g., *Il6*) [9, 11, 21]. RT-qPCR was therefore used to assess the impact of PEX treatment on expression of the pro-inflammatory cytokines *Il6* and *Il1b* as well as the chemokines *Cxcl1* and *Cxcl10*. RT-qPCR did not reliably detect expression of *Il6*, *Il1b*, *Cxcl1*, and *Cxcl10* in either brain region in WT mice regardless of treatment status (data not shown). *Il6* and *Il1b* expression was also not detected in the hippocampus of AxD mice (data not shown). In the olfactory bulb of AxD mice, *Il6* expression was reduced by drug treatment (Fig. 6a) while *Il1b* expression was not impacted (Fig. 6b). Similarly, *Cxcl1* and *Cxcl10* expression was not altered by PEX treatment in either brain region of AxD mice (Fig. 6c-f). In summary, PEX treatment reduced *Il6* expression in AxD mice; however, it did not impact expression of other cytokines/chemokines examined.

### Pexidartinib treatment does not alter T cell responses in AxD mice

**Fig. 7:**
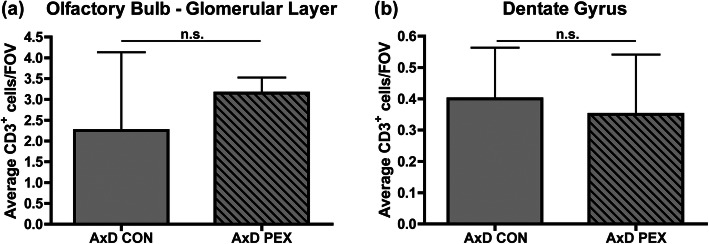
Pexidartinib treatment does not alter T cell responses in AxD mice. Immunofluorescence for CD3 was performed and CD3^+^ cells quantified in the olfactory bulb glomerular layer (**a**) and dentate gyrus (**b**) per FOV. Statistical analyses: unpaired two-sided *t* test, n.s., non-significant (*p*>0.05). *N* = 4 per group

T cell infiltration has been documented in both human patients with early onset AxD as well as a severely affected *GFAP*^Tg^;*Gfap*^R236H/+^ mouse model [21]. CD3 staining was therefore used to characterize T cell infiltration in the olfactory bulb glomerular layer and dentate gyrus. In both drug and control treated WT mice, very few CD3^+^ cells were observed in both regions and thus these groups were left out of further analyses. In contrast CD3^+^ cells were observed in both brain regions in AxD control mice, but their numbers were not impacted by PEX treatment (Fig. 7a and b, example images of CD3^+^ cells are shown in Additional figure 3a and b). This data indicates that T cell infiltration is not impacted by PEX treatment.

## Discussion

CSF1R inhibition has been tested for therapeutic efficacy in mouse models for other neurological disorders such as amyotrophic lateral sclerosis and multiple sclerosis [18, 20]. However, AxD is considered a primary astrocyte disorder and the role of macrophages in promoting disease phenotypes initiated by astrocyte dysfunction is not known. This report shows that upregulation of *Csf1* is a consistent feature of AxD models and that administration of the CSF1R inhibitor PEX to AxD model mice results in CNS macrophage depletion and elevated GFAP protein levels but did not lead to significant changes in many other disease phenotypes.

Elevated GFAP mRNA and protein levels have been observed in patients with AxD as well as in the *Gfap*^R236H/+^ mouse model [8, 9, 26]. In AxD mice, although PEX treatment caused only a slight increase in *Gfap* transcript levels in the olfactory bulb but not in the hippocampus, a large increase in GFAP protein levels was observed in both brain regions, although this did not translate into any differences in Rosenthal fiber accumulation. GFAP levels are known to be post-transcriptionally regulated in AxD [24, 25], and it is possible that PEX influences this process. Another possibility is that macrophages could be phagocytosing and degrading material, including GFAP. Previous studies in wild-type mice have found that PEX increases GFAP mRNA (nearly 2×) and protein levels (nearly 4×) [5]. The results of the current study do not recapitulate these findings, as no significant increases in transcript or protein levels were observed in the olfactory bulb or hippocampus of WT-treated mice. This difference may be explained by treatment time, as the previous study focused on shorter times (7 days and 21 days) where the current study employed long-term treatment. The prior study also analyzed half brain hemispheres while the current study utilized dissected brain regions, and regional differences have been observed in both *Gfap* and GFAP levels across the WT mouse brain [15]*.*

Increased expression of additional markers of astrogliosis and stress response activation are common features across AxD mouse models, and are thought to be downstream of expression of mutant GFAP and/or GFAP accumulation [8–10, 12, 16]. Our data analyzing *Lcn2*, *Cryab*, *Nfe2l2*, and *Nqo1* expression indicate that PEX treatment has little impact on these processes, as the only significant difference was a slight (1.17×) increase in *Cryab* in the olfactory bulb. These results were unexpected due to the increases in GFAP levels that were observed upon PEX treatment. It is possible that increased GFAP protein may be incorporated into normal filaments or other non-toxic forms, or that the increase was not dramatic enough to exacerbate these disease phenotypes in a detectable manner. Current AxD mouse models do not recapitulate myelin deficits that are present in certain forms of human AxD [8], and we observed no differences in expression levels of transcripts encoding two myelin components, *Plp1* (proteolipid protein (myelin) 1) and *Mog* (myelin oligodendrocyte glycoprotein), in PEX-treated WT or AxD mice (Additional figure 4a-d). This indicates that PEX treatment and resulting GFAP increases also do not appear to promote de novo myelin phenotypes in AxD model mice.

Our studies indicate that PEX has complex actions on the inflammatory response that occurs in AxD, as it mitigates *Il6* expression but did not impact *Il1b*, *Cxcl1*, and *Cxcl10* expression. This data indicates that cells other than macrophages can produce cytokines/chemokines in AxD, which is in line with previous observations in the *GFAP*^Tg^;*Gfap*^R236H/+^ model [21]. Moreover, our data indicate that long-term PEX treatment does not cause a de novo inflammatory response in WT mice. We also found that PEX treatment did not reduce the numbers of T cells in AxD, indicating that reducing macrophage numbers is not sufficient to blunt T cell recruitment. Although a T cell response had been previously documented in human patients with early onset AxD and a severely affected mouse model for AxD [21], our study is the first to report the presence of T cells in the brains of the *Gfap*^R236H/+^ mouse model for AxD. Because *Gfap*^R236H/+^ model mice have milder phenotypes, this indicates that T cell recruitment may be a consistent feature in AxD. However, further studies will be required to specifically examine T cell recruitment in human patients with less severe forms of the disease, and to determine if T cells contribute to AxD phenotypes.

In summary, these studies demonstrate that PEX treatment of an AxD mouse model causes CNS macrophage depletion and increased GFAP protein levels but has minimal impacts on other disease phenotypes. However, our study cannot definitively rule out macrophages as a therapeutic target in AxD. For example, PEX is known to also inhibit receptor tyrosine kinases in the same family as CSF1R [4] and therefore it may have impacts outside of macrophage lineage cells. In addition, given what is known in other CNS diseases, resident microglia and infiltrating peripheral macrophages could have differential impacts on disease phenotypes in AxD. Therefore, additional studies will be required to fully explore the phenotype and roles of macrophages in AxD.

## Conclusions

The results of this study, especially the impacts on GFAP levels, do not support the use of PEX as a therapy for AxD. However, our study also highlights the complexity of the immune response in AxD, and additional studies will be required to determine if neuroinflammation is a therapeutic target for disease treatment.

## Supplementary Information

**Additional file 1: Additional Figure 1**: Representative images for IBA1 staining. Legend: Representative images for the IBA1 staining in the glomerular layer of the olfactory bulb (a) with a 20 μm scale bar and the dentate gyrus (b) with a 50 μm scale bar.

**Additional file 2: Additional Figure 2**: Representative Hematoxylin & Eosin images of Rosenthal fibers. Legend: Representative images of the olfactory bulb glomerular layer (a) and along the hippocampal fissure (b). Scale bars represent 25 μm, arrowheads indicate some examples of Rosenthal fibers.

**Additional file 3: Additional Figure 3**: Examples of CD3 immunostaining. Legend: Examples (arrowheads) of CD3^+^ cells in the olfactory bulb glomerular layer (a) and the dentate gyrus (b) of AxD mice. Scale bars represent 20 μm.

**Additional file 4: Additional Figure 4**: Pexidartinib treatment does not impact expression of *Plp1* and *Mog.* Legend: RT-qPCR for *Plp1* for the olfactory bulb (a) and hippocampus (b), *Mog* for the olfactory bulb (c) and hippocampus (d). Statistical analyses: One-way ANOVA with Bonferroni’s post-test for the indicated comparisons, n.s. = non-significant (*p*>0.05). N = 5-6 per group.

## Data Availability

The data from this manuscript are available from the corresponding author upon reasonable request.

## Abbreviations

AxD: Alexander disease
GFAP: Glial fibrillary acidic protein
CNS: Central nervous system
PEX: Pexidartinib
CSF1R: Colony-stimulating factor 1 receptor
IBA1: Ionized calcium-binding adaptor molecule 1
CSF1: Colony stimulating factor 1
PFA: Paraformaldehyde
PBS: Phosphate-buffered saline
MOG: Myelin oligodendrocyte glycoprotein
PLP 1: Proteolipid protein 1
CON: Control
WT: Wild-type
ELISA: Enzyme-linked immunosorbent assay
RT-qPCR: Real-time quantitative polymerase chain reaction
BSA: Bovine serum albumin
IL-34: Interleukin 34
IL-6: Interleukin 6
IL-1β: Interleukin 1β
NRF2: Nuclear factor erythroid 2-related factor
NQO1: NAD(P)H quinone dehydrogenase 1
CXCL1: Chemokine (C-X-C motif) ligand 1
CXCL10: Chemokine (C-X-C motif) ligand 10
LCN2: Lipocalin 2
CRYAB: Alpha-crystallin B
