# Supplementary figures and images for: Pexidartinib treatment in Alexander disease model mice reduces macrophage numbers and increases glial fibrillary acidic protein levels, yet has minimal impact on other disease phenotypes
Source: J Neuroinflammation. 2021 Mar 8;18:67. doi: 10.1186/s12974-021-02118-x (PMC7941726; doi:10.1186/s12974-021-02118-x)

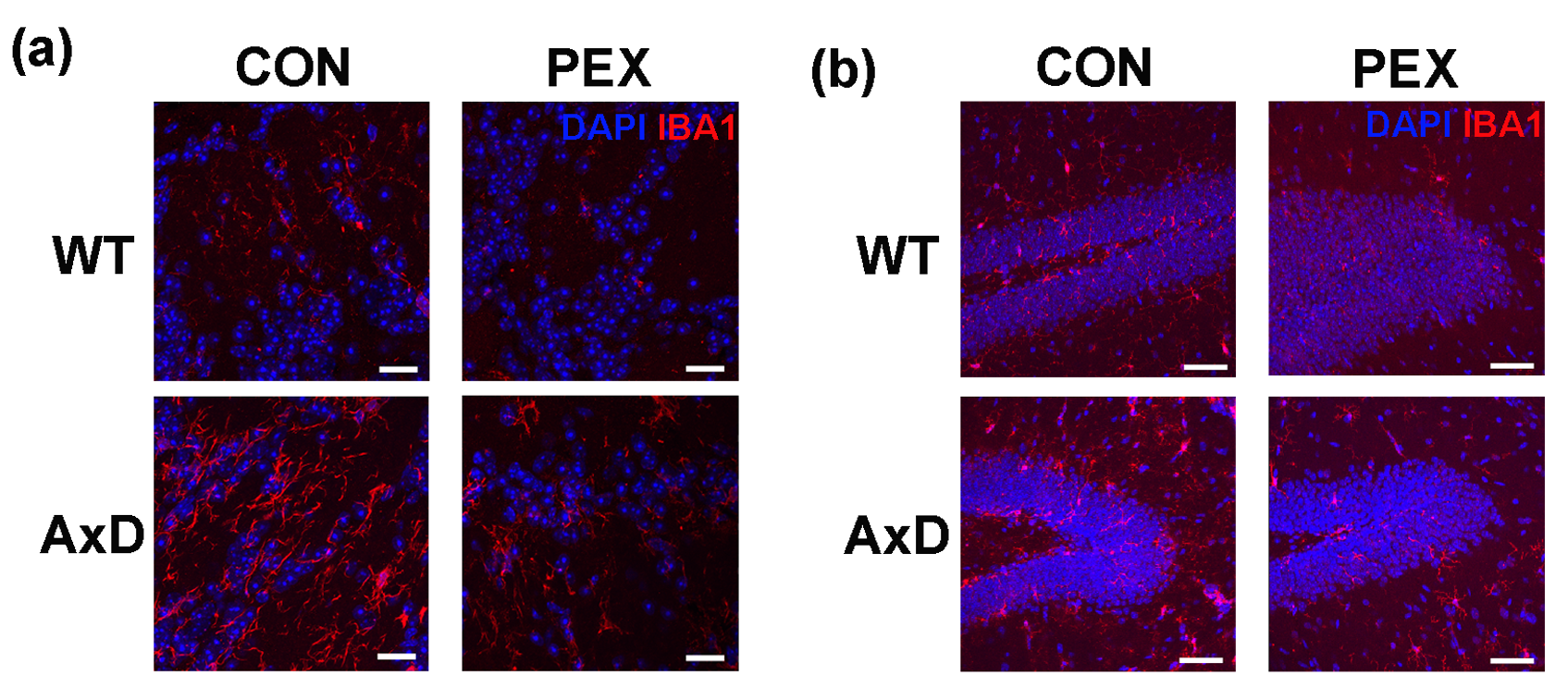

Supplement: Supplementary file 1 — Additional file 1: Additional Figure 1: Representative images for IBA1 staining. Legend: Representative images for the IBA1 staining in the glomerular layer of the olfactory bulb (a) with a 20 μm scale bar and the dentate gyrus (b) with a 50 μm scale bar. [file 12974_2021_2118_MOESM1_ESM.tif]

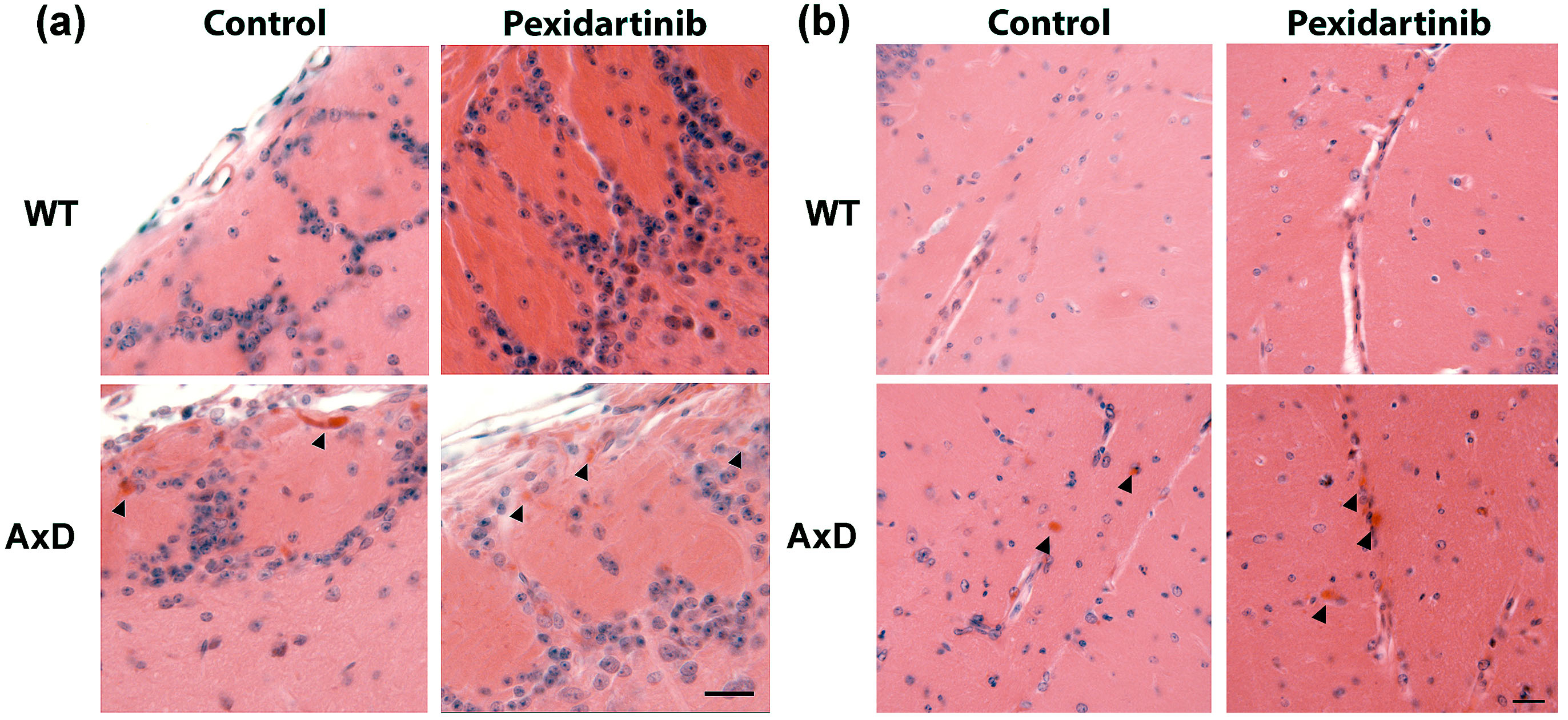

Supplement: Supplementary file 2 — Additional file 2: Additional Figure 2: Representative Hematoxylin & Eosin images of Rosenthal fibers. Legend: Representative images of the olfactory bulb glomerular layer (a) and along the hippocampal fissure (b). Scale bars represent 25 μm, arrowheads indicate some examples of Rosenthal fibers. [file 12974_2021_2118_MOESM2_ESM.jpg]

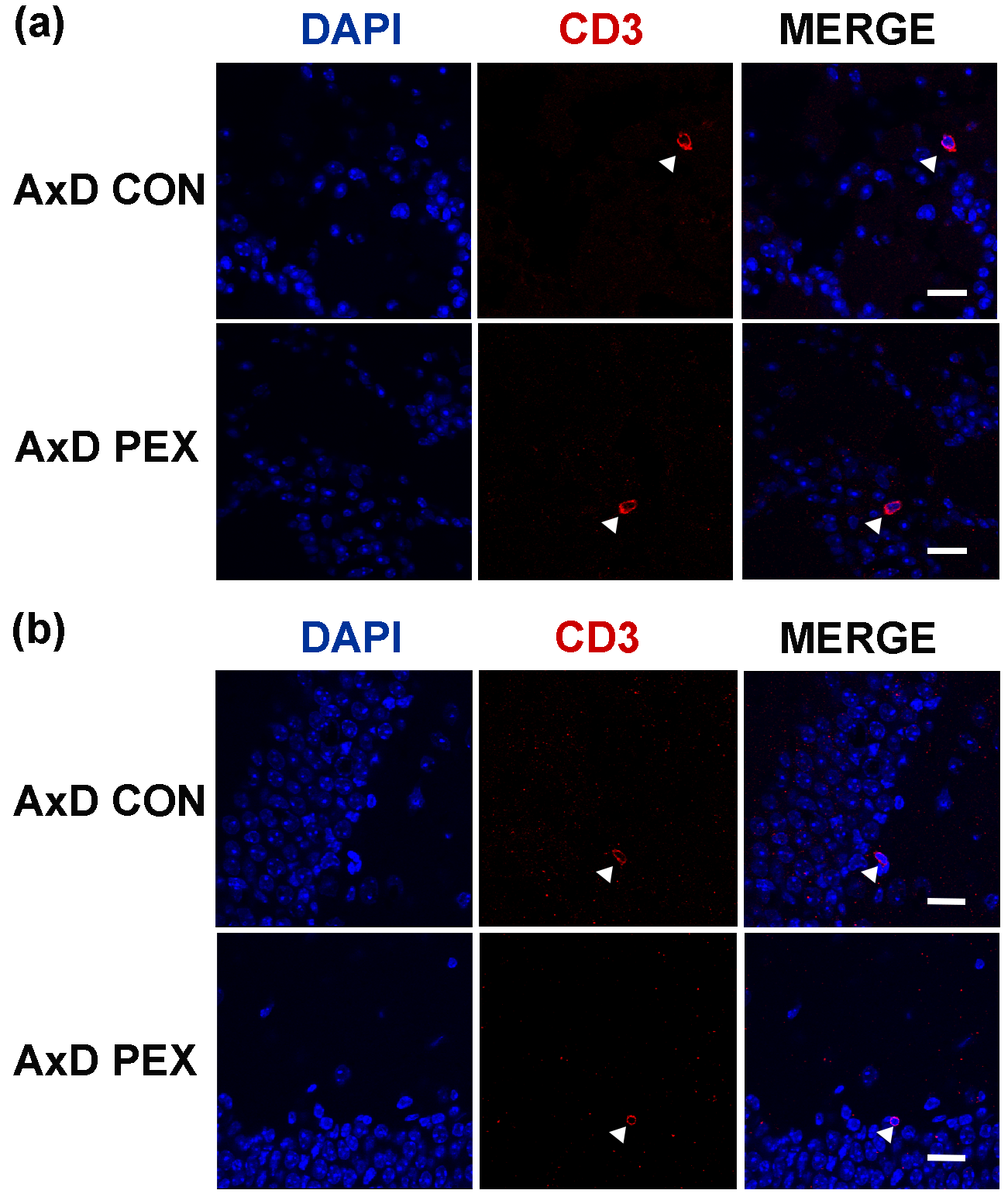

Supplement: Supplementary file 3 — Additional file 3: Additional Figure 3: Examples of CD3 immunostaining. Legend: Examples (arrowheads) of CD3+ cells in the olfactory bulb glomerular layer (a) and the dentate gyrus (b) of AxD mice. Scale bars represent 20 μm. [file 12974_2021_2118_MOESM3_ESM.tif]

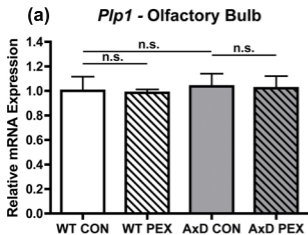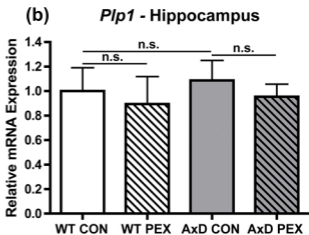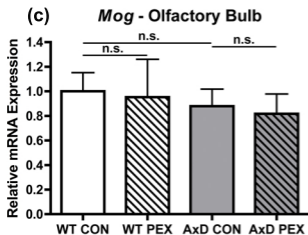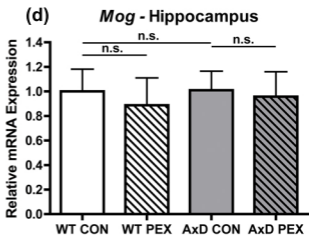

Supplement: Supplementary file 4 — Additional file 4: Additional Figure 4: Pexidartinib treatment does not impact expression of Plp1 and Mog. Legend: RT-qPCR for Plp1 for the olfactory bulb (a) and hippocampus (b), Mog for the olfactory bulb (c) and hippocampus (d). Statistical analyses: One-way ANOVA with Bonferroni’s post-test for the indicated comparisons, n.s. = non-significant (p>0.05). N = 5-6 per group. [file 12974_2021_2118_MOESM4_ESM.pdf]
